# Structures of Pathogenic Fungal FKBP12s Reveal Possible Self-Catalysis Function

**DOI:** 10.1128/mBio.00492-16

**Published:** 2016-04-26

**Authors:** Nam K. Tonthat, Praveen Rao Juvvadi, Hengshan Zhang, Soo Chan Lee, Ron Venters, Leonard Spicer, William J. Steinbach, Joseph Heitman, Maria A. Schumacher

**Affiliations:** a Department of Biochemistry, Duke University School of Medicine, Durham, North Carolina, USA; b Department of Pediatrics, Division of Pediatric Infectious Diseases, Duke University School of Medicine, Durham, North Carolina, USA; c Department of Molecular Genetics and Microbiology, Duke University School of Medicine, Durham, North Carolina, USA; d Department of Radiology, Duke University School of Medicine, Durham, North Carolina, USA

## Abstract

Invasive fungal infections remain difficult to treat and require novel targeting strategies. The 12-kDa FK506-binding protein (FKBP12) is a ubiquitously expressed peptidyl-prolyl isomerase with considerable homology between fungal pathogens and is thus a prime candidate for future targeting efforts to generate a panfungal strategy. Despite decades of research on FKBPs, their substrates and mechanisms of action remain unclear. Here we describe structural, biochemical, and *in vivo* analyses of FKBP12s from the pathogenic fungi *Candida albicans*, *Candida glabrata*, and *Aspergillus fumigatus*. Strikingly, multiple apo *A. fumigatus* and *C. albicans* FKBP12 crystal structures revealed a symmetric, intermolecular interaction involving the deep insertion of an active-site loop proline into the active-site pocket of an adjacent subunit. Such interactions have not been observed in previous FKBP structures. This finding indicates the possibility that this is a self-substrate interaction unique to the *A. fumigatus* and *C. albicans* fungal proteins that contain this central proline. Structures obtained with the proline in the *cis* and *trans* states provide more data in support of self-catalysis. Moreover, cysteine cross-linking experiments captured the interacting dimer, supporting the idea that it forms in solution. Finally, genetic studies exploring the impact of mutations altering the central proline and an adjacent residue provide evidence that any dimeric state formed *in vivo*, where FKBP12 concentrations are low, is transient. Taken together, these findings suggest a unique mechanism of self-substrate regulation by fungal FKBP12s, lending further novel understanding of this protein for future drug-targeting efforts.

## IMPORTANCE

FKBP12 is a *cis-trans* peptidyl-prolyl isomerase that plays key roles in cellular protein homeostasis. FKBP12s also bind the immunosuppressive drug FK506 to inhibit the phosphatase calcineurin (CaN). CaN is required for virulence of *A. fumigatus*, *C. albicans*, *C. glabrata*, and other deadly fungal pathogens, marking FKBP12 and CaN as potential broad-spectrum drug targets. Here we describe structures of fungal FKBP12s. Multiple apo *A. fumigatus* and *C. albicans* FKBP12 structures reveal the insertion of a proline, conspicuously conserved in these proteins, into the active sites of adjacent molecules. This suggests that these proteins might serve as their own substrates. Cysteine disulfide trapping experiments provide support for this self-interaction and hence possible intermolecular catalysis by these enzymes.

## INTRODUCTION

The 12-kDa FK506-binding protein (FKBP12) is a peptidyl-prolyl isomerase (PPIase) that is ubiquitously expressed in prokaryotic and eukaryotic cells and is a member of the FKBP PPIase superfamily (1–23). In addition to FKBPs, there are two additional classes of PPIases, the cyclophilins and the parvulins (10). PPIase proteins all catalyze the *cis/trans* isomerization of peptide bonds N terminal to proline residues in polypeptide chains. It is estimated that 5 to 7% of the proteins in eukaryotes that possess a peptidyl-prolyl bond acquire a *cis* conformation during folding. There is a large energy barrier of 14 to 24 kcal/mol between the *cis* and *trans* states, and hence, *cis/trans* isomerization is intrinsically slow and often represents the rate-limiting step in protein folding events (1–3). Therefore, PPIases, which catalyze this conformational change, play a central role in protein homeostasis in all cells (1–23).

In addition to its role as a PPIase, FKBP12 is a member of the immunophilin family and is the target of the immunosuppressive drugs FK506 and rapamycin (24–27). The FKBP12-FK506 complex binds the phosphatase calcineurin (CaN) and prevents it from dephosphorylating its downstream targets. CaN dephosphorylates the cytoplasmic component of the nuclear factor of activated T cells (NF-AT) transcription factor, which is necessary for interleukin-2 transcription and T-cell activation (28–32). As a result, inhibition of this key CaN function by FKBP12-FK506 leads to immunosuppression. CaN is also essential for the pathogenesis of some of the most deadly human fungal pathogens, including *Candida albicans*, *Candida glabrata*, and *Aspergillus fumigatus* (31). The fungal FKBP12 sequences are only 40-50% identical to their human counterpart, compared to the nearly identical interacting regions in the catalytic and regulatory domains of CaN proteins. Hence, fungal FKBP12s have emerged as potential targets for the development of broadly acting antifungal agents (21). The development of novel antifungal agents that specifically target the FKBP12 component in this pathway would be significantly aided by the structural elucidation of these proteins.

While there are currently no structures available for the fungal pathogen FKBP12s, multiple structures of FKBP12 and higher-molecular-weight FKBPs from humans and other organisms (including the model yeast *Saccharomyces cerevisiae*) have been determined in both their apo states and in complex with FK506 and rapamycin (27, 33, 34). These structures reveal that the FKBPs share the same overall fold that includes a five- to six-stranded β-sheet that wraps around an α-helix. Three extended loops, which have been called the 40s loop (between β2 and β3), the 50s loop (between β3′ and α1), and the 80s loop (between β4 and β5), surround a deep active-site pocket that binds both FK506 and rapamycin (18, 19). Despite the numerous structural, biochemical, and *in vivo* studies of FKBPs that have been carried out, their substrate specificities and catalytic mechanisms are still unclear. Indeed, there are no structures available for an FKBP12-substrate complex. The only structure that gives some insight into this enzyme-substrate interaction is the structure of the FKBP domain from *Plasmodium vivax* FKBP35 (*Pv*FKBP35) in complex with the unnatural tetrapeptide substrate succinyl-Ala–Leu–Pro–Phe–*p*-nitroanilide (35). However, the physiological relevance of this structure is unclear, as data showed that *Pv*FKBP35 is a monomer and in the crystal structure the substrate peptide is bound between two subunits of a crystallographic dimer.

To gain insight into the structure and function of the FKBP12s of *C. albicans*, *C. glabrata*, and *A. fumigatus*, we performed structural, biochemical, and genetic studies. Strikingly, the *C. albicans* and *A. fumigatus* FKBP12 apo structures all revealed the deep insertion of a proline located in the 80s loop into the ligand-binding pocket of a neighboring FKBP12 subunit. This interaction was not observed in the *C. glabrata* structure, which does not have the corresponding proline residue. Comparison of the apo structures with FKBP12-FK506 complexes reveals that this “self-substrate” overlaps the binding site of FK506. As FK506 is a transition state analog, these data suggest the possibility that these fungal FKBP12s might function as their own substrates. To test this hypothesis, we generated a single-cysteine mutant that was able to trap the self-interacting dimer, showing that it forms in solution. We also performed genetic studies to probe the effects of mutations in the 80s loop of *A. fumigatus* FKBP12. The combined studies support the possibility that the *A. fumigatus* and *C. albicans* FKBP12s may function as their own substrates, lending unique insight into this conserved and targetable fungal protein.

## RESULTS

### Structures of the apo *A. fumigatus* and *C. albicans* FKBP12s.

**FIG 1:**
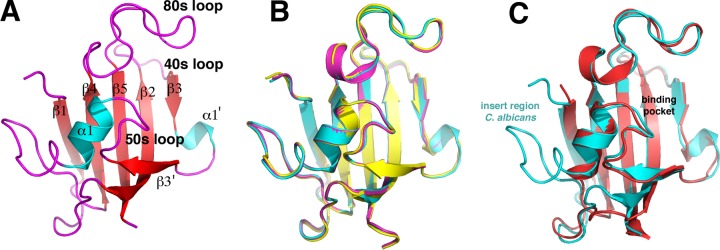
Crystal structures of *C. albicans* apo FKBP12. (A) Overall structure of the *C. albicans* FKBP12 apo form with the β-strand colored red, helices colored cyan, loops colored magenta, and secondary structural elements labeled. (B) Overlaid structures of the three crystal forms of *C. albicans* apo FKBP12 showing their overall identity. (C) Overlaid *C. albicans* (cyan) and human (red) FKBP12 structures. Labeled is the insert region that is not present in the human protein.

The *C. albicans* and *A. fumigatus* FKBP12s represent potential drug targets in treating invasive fungal infections. To gain insight into the structures and functions of these proteins, we determined their crystal structures. Three different crystal forms of the apo *C. albicans* FKBP12, to resolutions of 1.99, 2.3, and 2.4 Å, were obtained (Table 1). The FKBP12 subunit structures of the three crystal forms are essentially identical (root mean square deviations [RMSDs] of ~0.2 to 0.4 Å for superimpositions of corresponding C-α atoms) (Fig. 1A and B). Structural homology searches with DALI revealed that the *C. albicans* apo FKBP12 structures show significant structural homology to all FKBP12s, with the strongest similarity to the human protein (Protein Data Bank [PDB] code 1d7h); superimposition of corresponding C-α atoms of the *C. albicans* and human FKBP12 structures resulted in an RMSD of 1.02 Å (Fig. 1C). Like other FKBP12 structures, the *C. albicans* protein harbors the typical core FKBP12 structure, which consists of a five-stranded β-sheet wrapped about the central helix. In addition, *C. albicans* FKBP12 has an insert of 12 residues between α1 and β4 (Fig. 1A and C). Despite the extra residues in the insert region of the *C. albicans* structure, it contains the same pocket as other FKBPs, which is framed by the 40s, 50s, and 80s loops (Fig. 1C).

**TABLE 1:** Data collection and refinement statistics for *C. albicans* FKBP12 structures

Parameter	*C. albicans* FKBP12 apo form:			*C. albicans* FKBP12(P104G)-FK506	Apo *C. albicans* FKBP12(P104G)
	1	2	3		
Space group	C2	P2_1_2_1_2	P1	C2	P2_1_
Unit cell dimensions					
*a*, *b*, *c* (Å)	99.5, 43.0, 70.7	35.8, 80.7, 94.9	35.9, 38.6, 50.4	115.4, 84.2, 116.7	51.2, 44.0, 56.99
α = β = γ (°)	90.0, 128.2, 90.0	90.0, 90.0, 90.0	78.6, 69.7, 79.3	90.0, 109.8, 90.0	90.0, 115.0, 90.0
Resolution (Å)	26.0-2.4	40.9-2.3	63.8-1.99	26.1-2.9	45.3-2.0
*R*_merge_ (%)	10.1 (19.7)^a^	6.4 (55.9)	9.1 (31.2)	12.4 (38.7)	4.5 (17.3)
*I*/σ‹*I*›	13.2 (6.3)	23.6 (2.0)	5.8 (2.0)	10.7 (2.1)	20.8 (2.2)
Completeness (%)	98.2 (93.8)	98.8 (92.3)	85.2 (85.2)	84.3 (37.6)	99.0 (89.8)
Redundancy	3.3 (2.4)	3.0 (2.2)	1.6 (1.6)	2.8 (1.4)	3.3 (2.9)
Refinement					
Resolution range (Å)	25.96–2.4	40.9–2.3	63.8–1.99	26.1–2.9	45.3–2.0
*R*_work_/*R*_free_ (%)	17.7/22.2	17.9/22.8	22.5/25.4	24.4/28.5	18.0/19.5
No. of atoms					
Protein	1,733	1,808	2,056	6,769	3,788
Water	129	67	166	64	161
RMSD					
Bond length (Å)	0.004	0.008	0.002	0.008	0.003
Bond angle (°)	0.86	1.42	1.29	1.59	0.88

a Values in parentheses are for the highest-resolution shell.

### *C. albicans* apo FKBP12 structures reveal intermolecular contacts between active sites.

**FIG 2:**
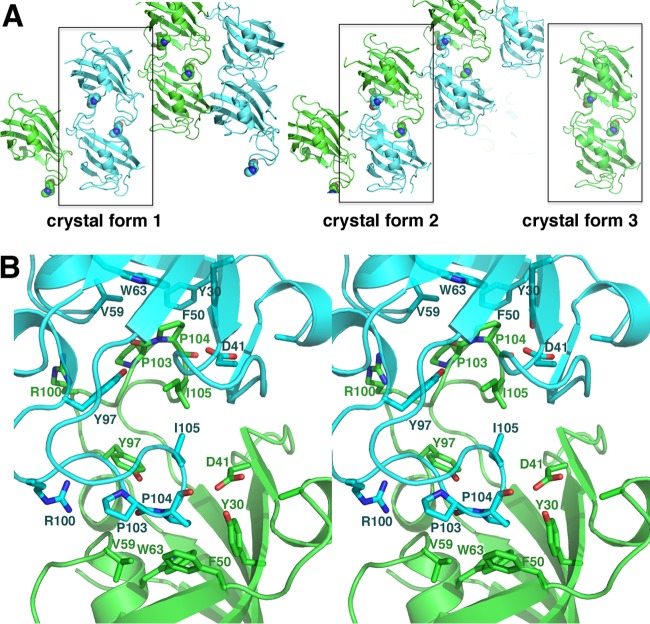
Intermolecular active-site cross contacts revealed in *C. albicans* apo structures. (A) Packing of the three crystal forms of *C. albicans* apo FKBP12. In all cases, an intermolecular contact is formed in which the 80s loop is inserted into the active site of an adjacent molecule. The interaction is symmetric, and hence, each dimer has two “substrate” interactions. Pro104, which is located at the tip of the 80s loop and shown as a CPK model, is the most deeply inserted side chain, and it contacts key residues shown to be important for FKBP12 PPIase activity. (B) Stereo view of the two-fold related 80s loop active-site interactions in the *C. albicans* apo FKBP12 structures. Notably, a previous study (36) showed that residues Asp41, Val59, and Tyr97 are critical for FKBP12 PPIase activity. In the structures, these residues directly contact or form part of the pocket for binding of the Pro104 side chain. Notably, the Asp41 side chain makes hydrogen bonds with the Pro104 carbonyl oxygen in each pocket.

While the *C. albicans* apo FKBP12 structure is very similar to previously determined FKBP12 structures, an unexpected finding that emerged from analysis of the crystal packing was that despite being obtained under very different crystallization conditions with distinct crystal lattices, all three *C. albicans* apo FKBP12 structures displayed the same intermolecular interactions between subunits (Fig. 2A). What is notable about this interaction is that the 80s loop of one subunit docks into the active site of the adjacent subunit and vice versa, suggesting that the loop may act as a substrate. Indeed, Pro104, which is located at the tip of the loop, is inserted most deeply into the pocket and is surrounded by residues that have been implicated, via mutagenesis, to be involved in FKBP PPIase activity (36, 37). Three residues, in particular, were implicated as important for FKBP12 catalysis and correspond to Val59, Asp41, and Tyr97 in the *C. albicans* protein (Fig. 2B). These residues all make direct contacts with Pro104, which rests within an aromatic cleft formed by Tyr30, Phe50, and Trp63 (Fig. 2B). Notably, the interface in the *C. albicans* FKBP12-FKBP12 interaction is minimal (burying 592 Å^2^ from solvent), which suggests that, like known substrate-enzyme complexes, the proteins form a weak interaction; typical dimer interfaces bury >2,000 Å^2^ of surface from solvent. Although most of the contacts that hold the loop in position in the active site are with the central Pro104, additional hydrophobic contacts with Ile102, Pro103, and Ile105 are provided, while Arg100 makes hydrogen bonds with the carbonyl oxygens at the edge of the pocket.

**FIG 3:**
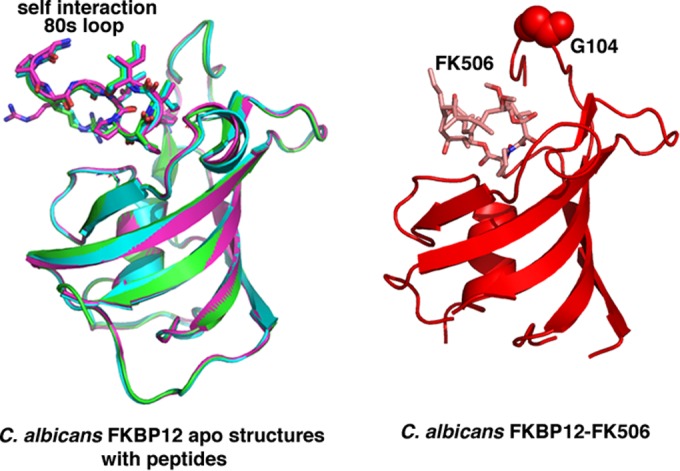
The 80s loop of the *C. albicans* FKBP12 apo structures binds in the same pocket as the transition state mimic FK506. On the left are overlays of the three *C. albicans* apo FKBP12 structures with the 80s loop “substrate” included. On the right is the structure of *C. albicans* FKBP12-FK506. The 80s loops from the apo structures are inserted in the same way and in the same pocket as the transition state mimic FK506 (right).

Studies have indicated that the immunosuppressant FK506 functions as a transition state mimic when bound to FKBPs (19). Comparison of the *C. albicans* FKBP12 apo structure with FKBP12-FK506 structures reveals that the 80s loop binds in the same pocket as FK506. However, to confirm that this is indeed the catalytic pocket in the *C. albicans* protein, we next sought to determine the *C. albicans* FKBP12-FK506 structure. Interestingly, initial attempts to obtain crystals of the protein-FK506 complex failed, as the intermolecular interaction appeared to be strongly favored, leading to the production of the same apo crystal forms. Thus, we generated a P104G mutation in *C. albicans* FKBP12 and crystallized and obtained the apo FKBP12(P104G) structure to 2.0 Å (Table 1). Strikingly, this structure did not harbor the self-catalysis-like interaction (see Fig. S1 in the supplemental material). We next used the FKBP12(P104G) protein to obtain the structure bound to FK506 (Table 1). The structure revealed that, similar to other FKBP12-FK506 complexes, the immunosuppressant binds in the active-site pocket, surrounded by the 40s, 50s, and 80s loops. Comparison of this structure with the apo *C. albicans* structures with bound self-substrates revealed that the 80s loop that is inserted into the active site of the adjacent subunit binds in precisely the same location as the FK506 substrate/transition state homolog (Fig. 3).

### *A. fumigatus* FKBP12 structures: self-catalysis captured?

**FIG 4:**
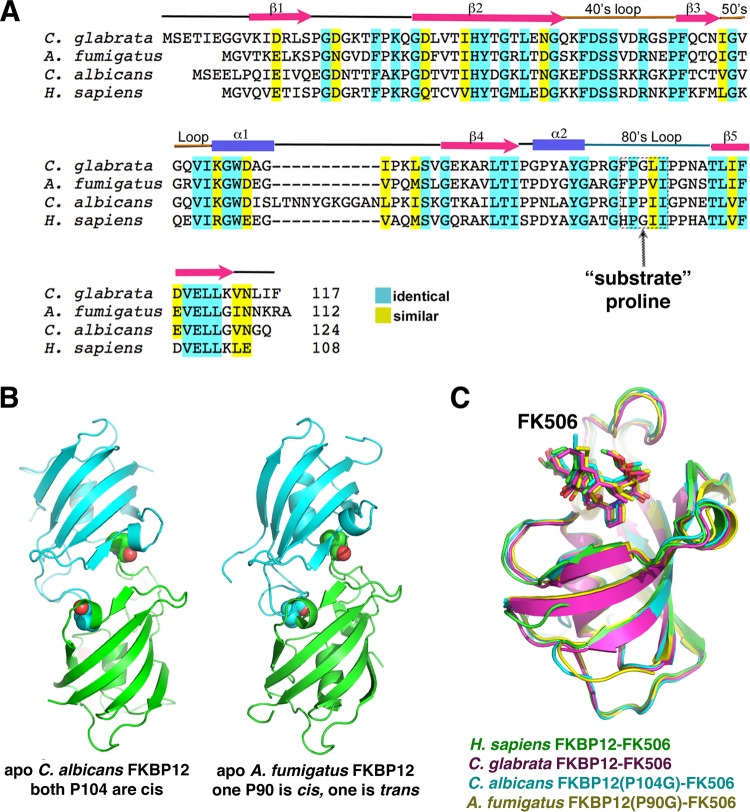
Alignment of FKBP12 sequences. The 80s loop proline is conserved in *C. albicans* and *A. fumigatus* FKBP12 but not in *C. glabrata*, and *H. sapiens* FKBP12s. (A) Sequence alignments of the FKBP12s of *C. albicans*, *A. fumigatus*, *C. glabrata*, and *H. sapiens*. Secondary structural elements are shown above the sequence. Identical and highly conserved residues are highlighted in cyan and yellow, respectively. (B) The *A. fumigatus* FKBP12 apo structure shows the same self-interaction as *C. albicans* FKBP12. Shown side by side are the self-interacting dimers of *A. fumigatus* and *C. albicans*. The proline corresponding to *C. albicans* Pro104 is Pro90 in *A. fumigatus* FKBP12, and interestingly, the *A. fumigatus* structure shows one subunit with Pro90 in the *cis* conformation and one with Pro90 in the *trans* conformation. (C) Superimpositions of the FKBP12-FK506 structures of *H. sapiens*, *C. albicans*, *C. glabrata*, and *A. fumigatus*, all showing the identical mode of FK506 binding.

Our data thus far revealed that *C. albicans* FKBP12 contains a proline residue at the tip of its 80s loop, Pro104, that, in the apo form, is inserted into the active site of a neighboring subunit. Sequence alignments revealed that *A. fumigatus* FKBP12 contains the same proline residue in its 80s loop that makes intermolecular interactions in the *C. albicans* protein (Table 2; Fig. 4A). Thus, to deduce if *A. fumigatus* FKBP12 might form a similar self-substrate-like interaction, we crystallized and determined the structure of the apo form of the protein. Remarkably, we observed the same insertion of the 80s loop of each subunit into the active site of the other subunit (Fig. 4B). As for *C. albicans* FKBP12, we also had difficulty obtaining the structure of the *A. fumigatus* wild-type (WT) protein in complex with FK506. Thus, we generated the analogous mutation, P90G, and used that protein to obtain the FKBP12(P90G)-FK506 structure. As expected the FK506 molecule is bound in the same active-site pocket of *A. fumigatus* FKBP12 as in other FKBP12-FK506 complex structures (Fig. 4C). Interestingly, unlike the *C. albicans* apo FKBP12 structures, which were all captured with Pro104 in the *cis* conformation, in the *A. fumigatus* structure, Pro90 in one subunit was bound in the *cis* conformation and Pro90 in the other was bound in the *trans* state, thus potentially providing a snapshot of self-catalysis (Fig. 4B).

**TABLE 2:** Data collection and refinement statistics for *A. fumigatus* FKBP12 structures

Parameter	Apo *A. fumigatus* FKBP12	*A. fumigatus* FKBP12(P90G)-FK506	*A. fumigatus* FKBP12(V91C) dimer
Space group	P2_1_2_1_2_1_	P2_1_2_1_2_1_	P6_5_
Cell dimensions			
*a*, *b*, *c* (Å)	44.8, 50.8, 103.0	37.0, 51.1 55.6	136.8, 136.8, 33.0
α, β, γ (°)	90.0, 90.0, 90.0	90.0, 90.0, 90.0	90.0, 90.0, 120.0
Resolution (Å)	45.6–2.3	37.6–2.0	44.8–3.2
*R*_merge_ (%)	6.9 (36.6)^a^	5.7 (19.5)	10.4 (56.9)
*I*/σ‹*I*›	18.5 (3.1)	19.7 (1.8)	8.9 (1.8)
Completeness (%)	98.7 (91.2)	90.3 (99.1)	99.9 (98.6)
Redundancy	4.2 (2.6)	5.2 (5.2)	4.6 (3.6)
Refinement			
Resolution (Å)	45.6–2.3	37.6–2.0	44.8–3.2
*R*_work_/*R*_free_ (%)	20.5/25.8	16.6/22.5	20.1/26.9
No. of atoms			
Protein	1,706	862	1,692
Water	115	147	0
RMSD			
Bond length (Å)	0.002	0.007	0.011
Bond angle (°)	0.72	1.22	2.09

a Values in parentheses are for the highest-resolution shell.

### *C*. *glabrata* FKBP12 structures.

Sequence alignments of FKBP12s from various fungal pathogens revealed that only the *A. fumigatus* and *C. albicans* proteins contain a central proline in the 80s loop. Another clinically important and often antifungal-resistant *Candida* species, *C. glabrata*, contains a glycine at this location, suggesting that this protein would not form the intermolecular interaction. To test this prediction, we determined the structures of *C. glabrata* FKBP12 in its apo and FK506-bound states (Table 3). Examination of the packing revealed a close contact between the 80s loop of one subunit and an adjacent molecule in the apo crystals. However, here the 80s loop is not inserted into the active site of the neighboring molecule. Hence, as predicted, the *C. glabrata* FKBP12 structure shows no evidence of an intermolecular self-catalysis-like binding event (see Fig. S2 in the supplemental material). However, to ensure that it utilizes the same active site, we obtained the structure in the presence of FK506 to 1.3 Å. As this structure clearly shows, FK506 binds to the active-site pocket by using a binding mode identical to that used by *C. albicans*, *A. fumigatus*, and other FKBP12s, including human FKBP12 (Fig. 4C).

**TABLE 3:** Data collection and refinement statistics for *C. glabrata* FKBP12 structures

Parameter	Apo *C. glabrata* FKBP12	*C. glabrata* FKBP12-FK506
Space group	I422	P6_3_
Cell dimensions		
*a*, *b*, *c* (Å)	76.44, 76.44, 87.0	77.8, 77.8, 53.6
α, β, γ (°)	90.0, 90.0, 90.0	90.0, 90.0, 120.0
Resolution (Å)	43.5–2.65	42.0–1.30
*R*_merge_ (%)	12.0 (36.6)^a^	4.6 (41.4)
*I*/σ‹*I*›	11.8 (2.8)	11.7 (1.7)
Completeness (%)	98.7 (47.3)	97.7 (93.3)
Redundancy	3.3 (2.2)	3.6 (2.0)
Refinement		
Resolution (Å)	43.5–2.65	42.0–1.30
*R*_work_/*R*_free_ (%)	21.4/26.0	15.3/16.4
No. of atoms		
Protein	873	873
Water	17	246
RMSD		
Bond length (Å)	0.003	0.025
Bond angle (°)	0.73	2.14

a Values in parentheses are for the highest-resolution shell.

### Disulfide cross-linking captures the FKBP12 intermolecular interaction.

**FIG 5:**
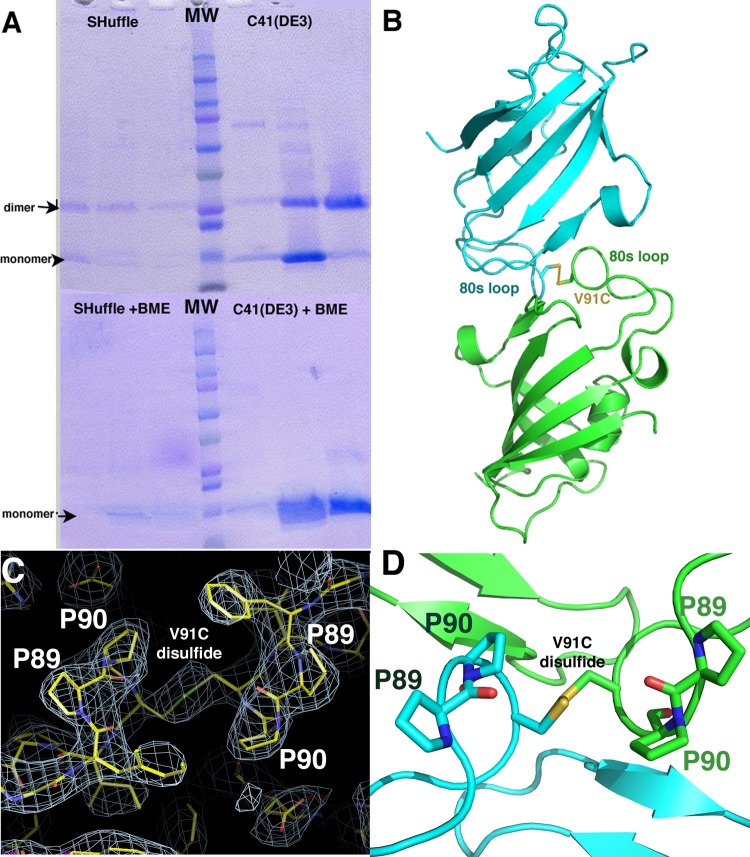
*A. fumigatus* FKBP12(V91C) mutation captures the intermolecular contact. (A) SDS-PAGE purification of FKBP12(V91C) from both SHuffle and C41(DE3) cells. Dimers are evident in the gel run without a reducing agent. Addition of a reducing agent leads to reduction of the disulfide bond and the proteins running as monomers. (B) Crystal structure of *A. fumigatus* FKBP12(V91C). (C) 2*F_o_*-*F_c_* electron density map contoured at 1 σ showing the region around the V91C disulfide bond and the Pro89-Pro90 bond, which adopts a twisted, *trans* conformation-like state. (D) Ribbon diagram showing a closeup of the Pro89-Pro90 region with the residues labeled. Note that the green-labeled Pro90 side chains appear distorted.

While every structure of the WT apo *A. fumigatus* and *C. albicans* FKBP12s revealed the same interproline interaction, even under very diverse crystallization conditions, it remained possible that these were all crystallization artifacts. Thus, to assess if this interaction occurs in solution, we made a single cysteine mutation, V91C, in the *A. fumigatus* protein to try to capture a disulfide cross-linked complex. This residue was chosen for mutation because the structure revealed that the V91 and V91′ (where the prime indicates the other subunit of the dimer) side chains are within <4.0 Å of each other and that when they are mutated to cysteines *in silico* they could form a disulfide bond in the intermolecular dimer. The *A. fumigatus* FKBP12(V91C) mutant was expressed in two *Escherichia coli* cell lines, C41(DE3) and SHuffle cells (see Materials and Methods). SHuffle cells constitutively express a chromosomal copy of the disulfide bond isomerase DsbC, which promotes disulfide bond formation in the cytoplasm. When the proteins were purified from both strains in the absence of a reducing agent and subjected to nonreducing SDS-PAGE, the samples revealed the presence of two species, which were consistent with FKBP12 monomers and dimers (Fig. 5A). While the protein was not as well expressed in SHuffle cells, the samples purified from these cells ran primarily as dimers. When a reducing agent (β-mercaptoethanol) was added, the proteins ran as monomers (Fig. 5A). However, to ensure that the dimeric species in the gel was not an impurity that was sensitive to the reducing agent, we performed mass spectrometry of the dimer and monomer bands. The results confirmed that both species contained the *A. fumigatus* FKBP12(V91C) protein (data not shown).

We next separated the V91C dimer from the monomer via size exclusion chromatography and crystallized and solved the structure of the dimer to 3.2 Å resolution. The structure revealed a disulfide cross-linked dimer. Interestingly, although revealing the same general dimer that we observed in the *A. fumigatus* and *C. albicans* apo FKBP12 structures, in the FKBP12(V91C) structure (Fig. 5B), Pro90 is slightly shifted from the active site and adopts a twisted conformation (Fig. 5C and D), suggesting that the structure may have captured an intermediate state of catalysis. In fact, the conformation of the Pro90 residues in the structure is closer to the *trans* than the *cis* state and overlays of the *trans* state of the protein to form an all-*trans* dimer revealed a clash. This finding suggests the intriguing possibility that part of the mechanism of prolyl isomerization by FKBPs is dictated by the conformation of the substrate during catalysis. Indeed, the *cis* and *trans* states lead to structural changes in the loops and one state may be more favorable for binding of the active-site pocket than the other, thus determining the progress of the reaction.

### Examination of the *A. fumigatus* FKBP12 interaction via Y2H analysis.

To next assess if we could detect the *A. fumigatus* FKBP12 self-interaction *in vivo*, we employed the yeast two-hybrid (Y2H) system. The pGADT7 and pGBKT7 vectors, which express the GAL4 activation domain (AD) and the DNA-binding domain (BD), respectively, were employed for these studies (see Materials and Methods; see Table S1 in the supplemental material). We utilized a specialized host strain (SMY4-1) derived from the Y2H host strain Y190 and rendered FK506 and rapamycin resistant by *fpr1::ADE2* and *TOR1-3* mutations that allow testing of the impact of these FKBP12 ligands on any interactions detected (38). *A. fumigatus* WT FKBP12, FKBP12(P90G), and FKBP12(V91C) were tested. The N or C termini of the FKBP12s were fused with the AD (e.g., FkbA-AD or AD-FkbA), and the N termini of the FKBP12s were fused with the BD (e.g., BD-FkbA) (see Table S1). Interactions were assessed in the presence or absence of FK506 (1 µg/ml) or rapamycin (1 µg/ml) (see Materials and Methods) (38). These experiments revealed no strong self-interactions between the FKBP12s, suggesting that the proteins did not form stable dimers in the environment of the *S. cerevisiae* nucleus (Table 4; see Fig. S3 in the supplemental material). However, this was not necessarily surprising, since we did not expect strong interactions, given the low *in vivo* concentration of FKBP12 in the cell compared to that used for crystallization. Indeed, enzyme-substrate interactions cannot be tight, as that would prevent product turnover.

**TABLE 4:** Interactions of FKBP12, FKBP12 variants, and the FRB domain determined by Y2H analysis

AD-gene fusion	BD-gene fusion	Interaction	Drug^b^
N termini^a^			
FKBP12	FRB	−	Rapamycin
FKBP12	FRB	−	
FKBP12(P90G)	FRB	+	Rapamycin
FKBP12(P90G)	FRB	−	
FKBP12(V91C)	FRB	−	Rapamycin
FKBP12(V91C)	FRB	−	
FRB	FKBP12	+	Rapamycin
FRB	FKBP12	−	
FRB	FKBP12(P90G)	+	Rapamycin
FRB	FKBP12(P90G)	−	
FRB	FKBP12(V91C)	+	Rapamycin
FRB	FKBP12(V91C)	−	
C termini^c^			
FKBP12	FRB	+	Rapamycin
FKBP12	FRB	−	
FKBP12(P90G)	FRB	+	Rapamycin
FKBP12(P90G)	FRB	−	
FKBP12(V91C)	FRB	+	Rapamycin
FKBP12(V91C)	FRB	−	
FKBP12	FKBP12	−	Rapamycin
FKBP12	FKBP12	−	FK506
FKBP12	FKBP12	−	
FKBP12(P90G)	FKBP12(P90G)	−	Rapamycin
FKBP12(P90G)	FKBP12(P90G)	−	FK506
FKBP12(P90G)	FKBP12(P90G)	−	
FKBP12(V91C)	FKBP12(V91C)	−	Rapamycin
FKBP12(V91C)	FKBP12(V91C)	−	FK506
FKBP12(V91C)	FKBP12(V91C)	−	

a N termini of FKBP12s were fused with the GAL4 AD.

b Both rapamycin and FK506 were used at 1 µg/ml.

c C termini of FKBP12s were fused with the GAL4 AD.

Interestingly, we did observe that there is potential variation in the interaction affinity between FKBP12 and its variants during control interactions with the FKBP12-rapamycin complex-binding (FRB) domain of the TOR protein. Specifically, when the N terminus of FKBP12 or FKBP12(V91C) was linked with the AD [AD-FkbA or AD-FkbA(V91C)], it did not interact with the FRB domain in the presence of rapamycin, in contrast to other fungal FKBP12s, which revealed a strong interaction with the FRB domain under these conditions (39, 40). On the other hand, *A. fumigatus* AD-FKBP12(P90G) did reveal an interaction with the FRB domain in the presence of rapamycin. These data suggest that the properties of *A. fumigatus* FKBP12 may be altered by changing the amino acid at the 90th residue from proline to glycine.

We do not think that the failure to observe AD-FkbA or AD-FkbA(V91C) binding to the FRB domain is due to complete occupation of the AD-FkbA active site by another AD-FkbA molecule forming the dimer, as we expect this type of self-interaction to be transient. Similarly, because the cytoplasm and nucleus are reducing environments, we do not think the FkbA(V91C) disulfide-linked dimer will be a predominant species under these conditions. It is possible that the conformation of the 80s loop in WT FkbA (or V91C) versus FkbA(P90G) influences the binding of the FKBP12-rapamycin complex to the FRB domain of the TOR protein.

### *A. fumigatus* FKBP12(P90G) and FKBP12(V91C) mutations confer FK506 resistance.

**FIG 6:**
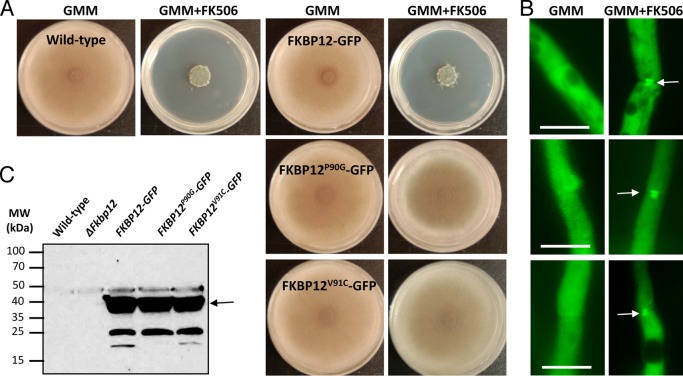
*In vivo* analysis of mutant *A. fumigatus* FKBP12s. (A) Radial growth of the WT strain (*akuB^KU80^*) and the strains expressing WT FKBP12-GFP and the mutated versions of FKBP12 [FKBP12(P90G) and FKBP12(V91C)] tagged with GFP were assessed after 5 days on GMM agar with or without supplementation with FK506 (100 ng/ml). Note the resistance of the FKBP12(P90G)- and FKBP12(V91C)-producing strains to FK506. (B) Strains expressing WT FKBP12-GFP and the mutated versions of FKBP12 [FKBP12(P90G) and FKBP12(V91C)] tagged with GFP were cultured in liquid GMM in the absence or presence of FK506 (100 ng/ml) on coverslips for 18 to 20 h and observed for the localization of FKBP12 by fluorescence microscopy. Note the complete cytosolic localization of FKBP12s in the absence of FK506. Arrows indicate the translocation of FKBP12s to the hyphal septa in the presence of FK506 indicative of their binding to CaN at the hyphal septa. Scale bar, 10 µm. (C) Western analysis performed with the anti-GFP polyclonal primary antibody and a peroxidase-labeled anti-rabbit IgG secondary antibody. The arrow indicates the ~37-kDa FKBP12-GFP fusion protein. The values on the left are molecular masses in kilodaltons.

On the basis of our structural and biochemical studies, the FKBP12(P90G) mutant would be predicted to be unable to self-catalyze, while the FKBP12(V91C) mutant might have altered target-binding properties. To test this, *A. fumigatus* strains expressing FKBP12(P90G) and FKBP12(V91C) tagged with enhanced green fluorescent protein (GFP) were generated and assessed for susceptibility to FK506 treatment (Fig. 6A to C). The stability of the respective mutated proteins was verified by Western analysis (Fig. 6C). As shown in Fig. 6A, both of the mutations induced relative FK506 resistance. FKBP12(V91C) exhibited greater resistance to FK506 than FKBP12(P90G) did (Fig. 6A; see Fig. S4 in the supplemental material), indicating a potential loss of affinity for CaN. In order to examine the binding of the mutated FKBP12s to CaN, we also screened for their intracellular localization in the absence or presence of FK506. In the absence of FK506, FKBP12(P90G) and FKBP12(V91C) localized evenly throughout the cytoplasm and also in the nuclei (Fig. 6B). Upon exposure to FK506, septal localization in a disc-like pattern, as noted earlier with CaN, was evident (Fig. 6B). Interestingly, however, both the FKBP12(P90G) and FKBP12(V91C) proteins were still able to bind to CaN at the septa but to a lesser extent than WT FKBP12. Furthermore, FKBP12(V91C) showed 40 to 50% less septal localization than WT FKBP12, confirming its lower affinity for CaN. These observations support the idea that the sequence and conformation of the 80s loop can influence fungal FKBP12-FK506 binding to CaN, in accordance with previous studies of human FKBP12-FK506 binding to CaN (41).

## DISCUSSION

The FKBP12s of the pathogenic fungi *A. fumigatus*, *C. albicans*, and *C. glabrata* have emerged as potential targets for drug design because of their role in the inhibition of CaN, a critical component of a cell signaling pathway essential for fungal pathogenesis (31). FKBP12 inhibits CaN by binding the immunosuppressant FK506. This FKBP12-FK506 complex then docks onto CaN, preventing it from binding substrates. Hence, the design of fungus-specific inhibitors of this complex would be greatly aided by structures of fungal FKBP12s in the presence or absence of FK506 and FK506-like compounds. Therefore, we undertook the determination of the structures of the *A. fumigatus*, *C. albicans*, and *C. glabrata* FKBP12s in their apo and FK506-bound forms. The apo structures of the *A. fumigatus* and *C. albicans* proteins all revealed the unexpected finding that one of the loops, the 80s loop, is inserted into the active-site pocket of an adjacent subunit. This finding led to the hypothesis that the *A. fumigatus* and *C. albicans* FKBP12s may function to isomerize their own proline residues. Support for this notion comes from previous mutagenesis studies (36) that revealed that the central hydrophobic pocket where the prolines from the 80s loop bind is indeed the location of the PPIase active site. However, despite the large number of FKBP structures solved to date, no structure of an FKBP bound to a substrate is currently available, which has left the details of the PPIase mechanism unclear and has limited structure-based targeting design efforts. Thus, if the *A. fumigatus* and *C. albicans* FKBP12 apo structures have captured a self-substrate interaction, this would be significant, as it would indicate that these represent the first FKBP-substrate complex structures. Support for this hypothesis was provided by the analysis of a cysteine mutant form of the *A. fumigatus* protein that trapped the interacting dimer.

The possibility that the 80s loop of the fungal proteins might function as a self-substrate would be of interest from a physiological standpoint, as studies have highlighted the 80s loop as a key region in the interactions of FKBP12s with other proteins (26). As *cis/trans* isomerization impacts the conformation of this loop, it would therefore significantly impact the functions of FKBP12 outside their PPIase activity. Indeed, FKBPs, which are ubiquitous molecules with homologs found in bacteria to higher eukaryotes, carry out a wide range of functions, including roles outside their PPIase activity (26). For example, studies with mice revealed that FKBP12 is critical for modulating the calcium release activity of both skeletal and cardiac ryanodine receptors (42). FKBP12 was also shown to be a regulator of the cell cycle through its effect on transforming growth factor beta receptor signaling (20). However, the specific functions of FKBP12 appear to vary dramatically between organisms. This diversity of FKBP functions has made analyzing the contributions of PPIase activity versus effector protein binding difficult and also indicates that the role of FKBP12 in each organism must be dissected individually. In this regard, it is currently not possible to obtain a clear genetic assessment of the hypothesis that the *A. fumigatus and C. albicans* FKBP12s act as their own substrates because the cellular roles of these proteins in these fungi have yet to be elucidated. Therefore, further studies are needed to deduce the potential importance of *A. fumigatus* and *C. albicans* FKBP12 self-catalysis and its possible effects on isomerization targets and interacting proteins and their impacts on resultant cellular pathways.

## MATERIALS AND METHODS

### Protein expression and purification.

Genes encoding the *C. albicans* and *A. fumigatus* FKBP12s that were codon optimized for *E. coli* expression were purchased from the GenScript Corporation (Piscataway, NJ) and subcloned into pET15b such that a hexahistidine tag (His tag), cleavable by thrombin, was added to the N termini. The proteins were expressed in *E. coli* BL21(DE3) cells. For protein expression, cells were grown to an optical density at 600 nm of ~0.6 at 37°C. At that time, the temperature was reduced to 15°C and isopropyl-β-d-thiogalactopyranoside was added to a final concentration of 1 mM. The cultures were allowed to grow for an additional 14 to 16 h, and the cells were pelleted and lysed in purification buffer (300 mM NaCl, 20 mM Tris-HCl [pH 7.5], 20 mM imidazole). The proteins were purified from the soluble fraction of the lysate via Ni-nitrilotriacetic acid (NTA) chromatography. Fractions containing FKBP12 were pooled and treated with thrombin at 4°C for 14 to 16 h to remove the N-terminal His tag. The digested sample was then applied to an Ni-NTA column, and the flowthrough was collected and loaded onto a Superdex 75 size exclusion column (GE Healthcare) equilibrated in elution buffer (150 mM NaCl, 20 mM Tris-HCl [pH 7.5]). The fractions containing pure protein were pooled and concentrated for biochemical studies and crystallization.

### Crystallization and determination of the structures of *A. fumigatus* and *C. albicans* FKBP12 apo and FK506 complexes.

*C. albicans* FKBP12 was crystallized in three different space groups, P1, P2_1_2_1_2, and C2, by hanging-drop vapor diffusion. The P1 crystal form was obtained by using a mixture of 25% polyethylene glycol (PEG) 3000, 0.1 M NaCl, and 0.1 M Tris-HCl (pH 8.0) as a crystallization reagent. The P2_1_2_1_2 form grew in a solution of 2 M ammonium sulfate and 0.1 M citrate (pH 5.5), and the C2 crystal form was crystallized in a mixture of 25% PEG 8000, 0.1 M sodium acetate (pH 4.5), and 0.2 M lithium sulfate. Each hanging drop contained a 1:1 ratio of ~10 mg/ml protein to crystallization reagent. The crystallization reagent supplemented with 25% glycerol was used as a cryosolvent. For cryopreservation, the crystals were dipped for several seconds in the cryosolvent prior to their placement in the liquid nitrogen stream. The *C. albicans* FKBP12(P104G)-FK506 complex crystallized in the P2_1_ space group in a mixture of 2 M ammonium sulfate, 0.1 M *N*-cyclohexyl-3-aminopropanesulfonic acid (CAPS)-NaOH (pH 10.5), and 0.2 M lithium sulfate. One millimole of FK506 was added to the protein prior to crystallization. A solution of 4 M ammonium sulfate was used as a cryoprotectant. *A. fumigatus* FKBP12 at a concentration of 20 mg/ml was crystallized in the P2_1_2_1_2_1_ space group in a mixture of 2 M ammonium sulfate, 0.1 M Tris-HCl (pH 7.0), and 0.2 M lithium sulfate. A 4 M ammonium sulfate solution was used as a cryoprotectant. *A. fumigatus* FKBP12(P90G)-FK506 crystals were obtained by mixing 20 mg/ml protein (with 1 mM FK506) with a solution containing 2 M lithium sulfate, 0.1 M Tris-HCl (pH 8.5), and 2% PEG 400 at a 1:1 ratio. *C. albicans* FKBP12(P104G) apo crystals were grown by mixing protein at 10 mg/ml 1:1 with a combination of 2 M ammonium sulfate, 0.1 M CAPS (pH 10.5), and 0.2 M lithium sulfate. The crystals could be cryopreserved straight from the drop. Data were collected at Advanced Light Source beamline 8.3.1 or Advanced Photon Source. Data were processed with HKL3000 or MOSFLM. Initial phases were obtained in each case by using molecular replacement (MolRep), starting with the human FKBP12 model (PDB code 2PPN). The *A. fumigatus* and *C. glabrata* apo structures were solved by using the *C. albicans* apo high-resolution structure as a starting model in MolRep. The FK506-bound structures were solved by using the requisite apo structures as starting models. All model building was carried out with COOT or O, and refinements were performed in PHENIX (42).

### Mutant *A. fumigatus* FKBP12(V91C) construction, disulfide cross-linking experiments, and determination of the structure of the cross-linked dimer.

Mutant *A. fumigatus* FKBP12(V91C) was made with the QuikChange kit. The pET15b plasmid expressing the mutant protein was transformed into C41(DE3) and SHuffle cells. The protein was expressed and purified in the same way as WT *A. fumigatus* FKBP12, except that no reducing agent was added at any step. The *A. fumigatus* FKBP12(V91C) dimer was separated from the monomer via size exclusion chromatography, and the His tag was cleaved before crystallization trials. The protein was concentrated to 30 mg/ml, and crystals were grown by mixing the protein solution 1:1 with a reservoir consisting of 1.5 M sodium citrate and 0.1 M sodium cacodylate (pH 6.5). Crystals grew within 3 h and were cryopreserved by being dipped for 1 s in a drop containing the crystallization solution supplemented with 25% glycerol. Data were collected and processed with MOSFLM. The structure was solved by MolRep with the *A. fumigatus* apo structure as a search model. The structure was refined with PHENIX (43).

### Construction of *A. fumigatus* FKBP12(P90G) and FKBP12(V91C) strains.

*A. fumigatus* WT strain *akuB^KU80^* was used for all transformation experiments and grown on glucose minimal medium (GMM) at 37°C (44). In certain experiments, GMM agar or RPMI liquid medium was supplemented with FK506 (0.1 to 10 µg/ml). All radial-growth experiments were repeated three times, each in triplicate. *E. coli* DH5α competent cells were used for subcloning. Site-directed mutagenesis of two FKBP12 residues (P90G and V91C) was performed with the primers listed in Table S2 in the supplemental material and the pUCGH-FKBP12 plasmid (45), consisting of 384 bp of the 637-bp *fkbp12* gene (*fkbp1*/Afu6g12170, http://www.aspergillusgenome.org) and ~1 kb of the *fkbp12* terminator sequence as the template. Briefly, in the first PCR, two fragments were amplified with complementary primers (with respective mutations) overlapping the *fkbp12* region to be mutated and the respective primers at the N and C termini of *fkbp12*. No stop codon was introduced at the C-terminal end of *fkbp12* to facilitate expression of the *gfp* fusion. Next, fusion PCR was done with equiproportional mixtures of the two PCR fragments as the templates, and the final mutated 384-bp *fkbp12* PCR fragment was amplified with primers at the N and C termini of *fkbp12* (see Table S2 in the supplemental material). Mutated *fkbp12* fragments were digested with KpnI and BamHI and cloned into the pUCGH-FKBP12 plasmid by replacing the 384-bp WT *fkbp12* PCR fragment to facilitate homologous integration. Mutated *fkbp12* genes were sequenced (see Table S2 for the primers used) to confirm the mutation and linearized with KpnI for homologous integration. Linearized constructs were transformed into *A. fumigatus akuB*^KU80^, and transformants were selected with hygromycin B (150 µg/ml) as previously described (46). Transformants were verified for homologous integration by PCR (see Table S2 and Fig. S5 in the supplemental material) and verified for accuracy of mutation by sequencing and fluorescence microscopy.

### Protein extraction and Western analysis.

*A. fumigatus* recombinant strains expressing respective WT and mutated forms of FKBP12-GFP fusion proteins were cultured in liquid GMM at 200 rpm for 24 h at 37°C. Crude extracts were prepared as previously described (47). Approximately 50 µg of protein electrophoresed on a 4 to 20% SDS-polyacrylamide gel was transferred onto a polyvinylidene difluoride membrane (Bio-Rad) and probed with a rabbit polyclonal antibody. The anti-GFP primary antibody (1 µg/ml; GenScript) and peroxidase-labeled rabbit anti-IgG (1:5,000; Rockland) secondary antibody. Detection was performed with SuperSignal West Pico Chemiluminescent Substrate (Thermo Scientific).

### Microscopy.

Conidia (10^4^) from the recombinant strains of *A. fumigatus* were inoculated into 5 ml of GMM, poured over a sterile coverslip (22 by 60 mm; no. 1), and placed in a sterile dish (60 by 15 mm). Cultures grown for 18 to 20 h at 37°C were observed by fluorescence microscopy with an Axioskop 2 plus microscope (Zeiss) equipped with AxioVision 4.6 imaging software.

### Y2H analysis.

The *A. fumigatus* fkbA gene and its variants, including *fkbA^P90G^* and *fkbA^V91C^* and the FRB domain-encoding gene were synthesized after codon optimization for *S. cerevisiae* expression (GenScript) and cloned into pGADT7 or pGBKT7 (Clontech Laboratories Inc.) for Y2H analysis. *S. cerevisiae* SMY4-1, which lacks the FKBP12 gene (*FPR1*), was used to examine the interactions. β-Galactosidase assays were performed as previously described (38, 39).

## SUPPLEMENTAL MATERIAL

Figure S1  Comparison of closest packing interactions of apo *C. albicans* FKBP12 and FKBP12(P104G) within crystals. One subunit is green, and the other is cyan. The active-site pocket of the green subunit is indicated by a dashed circle. In the WT apo structure, the proline residue (shown as a CPK model) is inserted into the active-site pocket of the adjoining subunit, while in the apo FKBP12(P104G) structure, Gly104 sits outside the pocket. Download Figure S1, TIF file, 0.3 MB

Figure S2  Comparison of the closest crystal packing interactions of the *C. albicans* apo FKBP12 (left) and *C. glabrata* apo FKBP12 (right) structures. Notably, although the *C. glabrata* structure has a close packing contact, it does not form the self-substrate interaction in the adjacent molecule’s active-site pocket (indicated by a dashed circle) that the *C. albicans* structure does. Download Figure S2, TIF file, 0.5 MB

Figure S3  Y2H analysis for interactions between *A. fumigatus* FKBP12 and its variants and the FRB domain. *A. fumigatus* FKBP12 and its variants did not exhibit interactions based on β-galactosidase activity in the presence or absence of FK506 (1 µg/ml). The asterisks indicate that dimeric interactions were observed *in vitro*. In the presence of rapamycin (rapa, 1 µg/ml), FkbA-AD and BD-FkbA interact with BD-FRB and AD-FRB, respectively. Download Figure S3, TIF file, 0.2 MB

Figure S4  Mutations conferring FK506 resistance. The WT strain (*akuB^KU80^*) and the FKBP12(P90G)- and FKBP12(V91C)-producing strains cultured in the absence (left panel) or presence of various concentrations (0.1 to 10 µg/ml) of FK506 for 24 h were visualized for growth. A total of 1 × 10^4^ conidia of each strain were inoculated into 200 µl of RPMI liquid medium in the absence or presence of FK506, and photographs (×10 magnification) were taken after 24 h of growth. Note the slightly greater resistance of the FKBP12(V91C)-producing strain than the FKBP12(P90G)-producing strain to FK506. Experiments were repeated three times, each in triplicate. Download Figure S4, TIF file, 0.8 MB

Figure S5  (A) Schematic representation of the genomic locus of the WT and recombinant *fkbp12* mutated strains. The partial coding sequence of the *A. fumigatus* fkbp12 gene was replaced with the mutated version (P90G or V91C) of *fkbp12* DNA fused to the *gfp* sequence at its C terminus with the hygromycin B resistance marker gene by homologous recombination. (B, C) PCR analysis for verification of the proper integration of the *fkbp12-gfp* construct at its native locus. Primers Fkbp12-F and pUCGH-R (indicated by arrows) were used to amplify the 750-bp PCR fragments from the recombinant strains (P90G and V91C transformants). Primers Hyg-Screen-F and Fkbp12-term-flank-R pUCGH-R (indicated by arrows) were used to amplify the 1,209-bp PCR fragments from the recombinant strains (P90G and V91C transformants). Download Figure S5, TIF file, 0.3 MB

Table S1  Plasmids and strains used for Y2H analysis. Table S1, DOC file, 0.1 MB

Table S2  Primers used to generate mutated FKBP12-GFP-producing strains. Table S2, DOC file, 0.03 MB
