# Supplementary figures and images for: Structures of Pathogenic Fungal FKBP12s Reveal Possible Self-Catalysis Function
Source: mBio. 2016 Apr 26;7(2):e00492-16. doi: 10.1128/mBio.00492-16 (PMC4850266; doi:10.1128/mBio.00492-16)

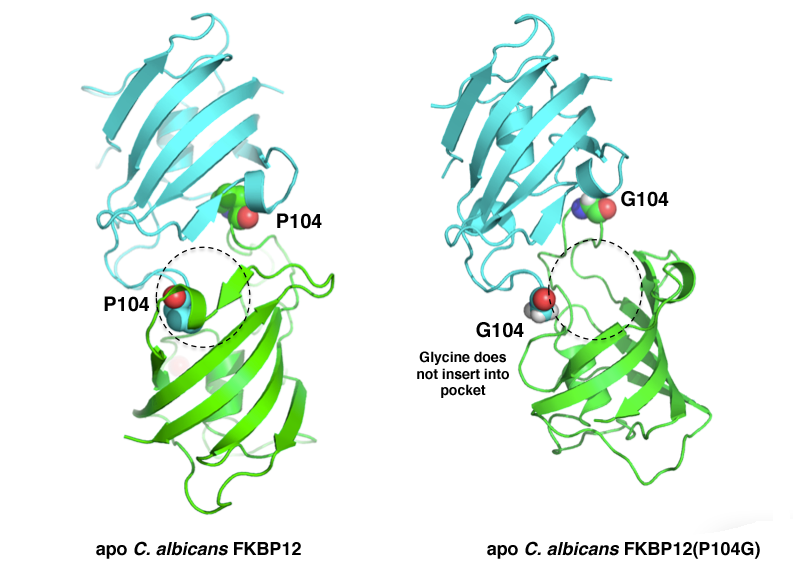

Supplement: Figure S1 — Comparison of closest packing interactions of apo C. albicans FKBP12 and FKBP12(P104G) within crystals. One subunit is green, and the other is cyan. The active-site pocket of the green subunit is indicated by a dashed circle. In the WT apo structure, the proline residue (shown as a CPK model) is inserted into the active-site pocket of the adjoining subunit, while in the apo FKBP12(P104G) structure, Gly104 sits outside the pocket. Download [file mbo002162791sf1.tif]

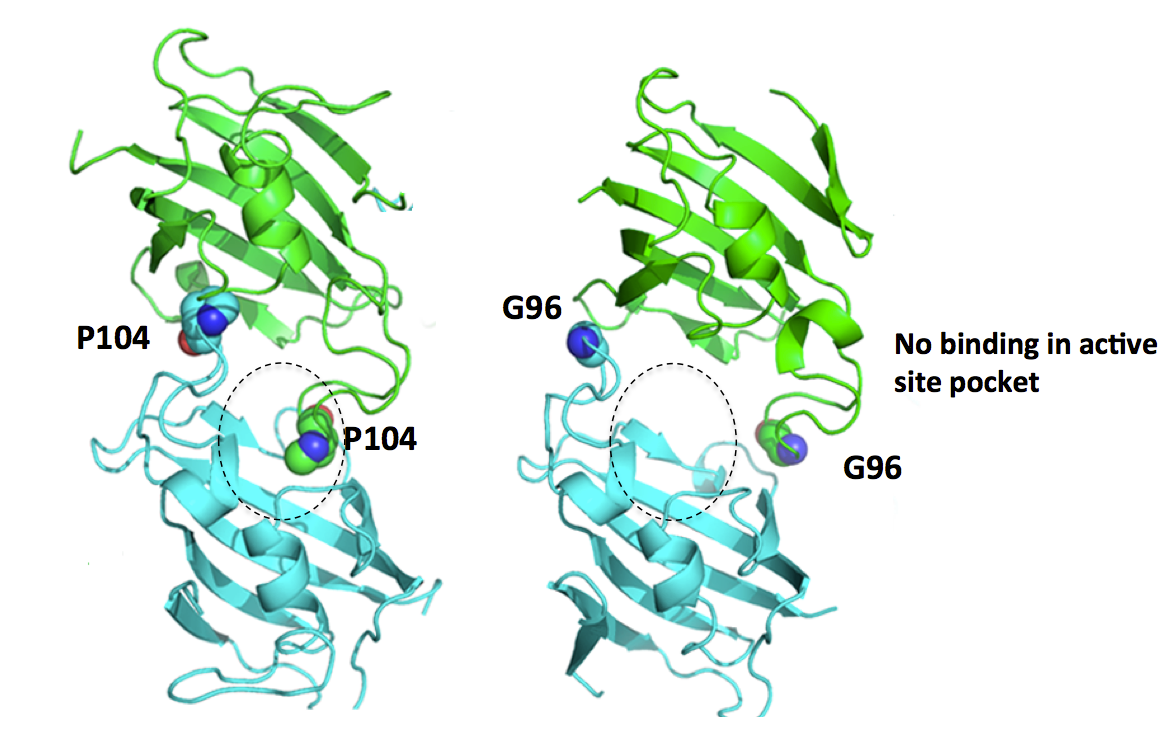

Supplement: Figure S2 — Comparison of the closest crystal packing interactions of the C. albicans apo FKBP12 (left) and C. glabrata apo FKBP12 (right) structures. Notably, although the C. glabrata structure has a close packing contact, it does not form the self-substrate interaction in the adjacent molecule’s active-site pocket (indicated by a dashed circle) that the C. albicans structure does. Download [file mbo002162791sf2.tif]

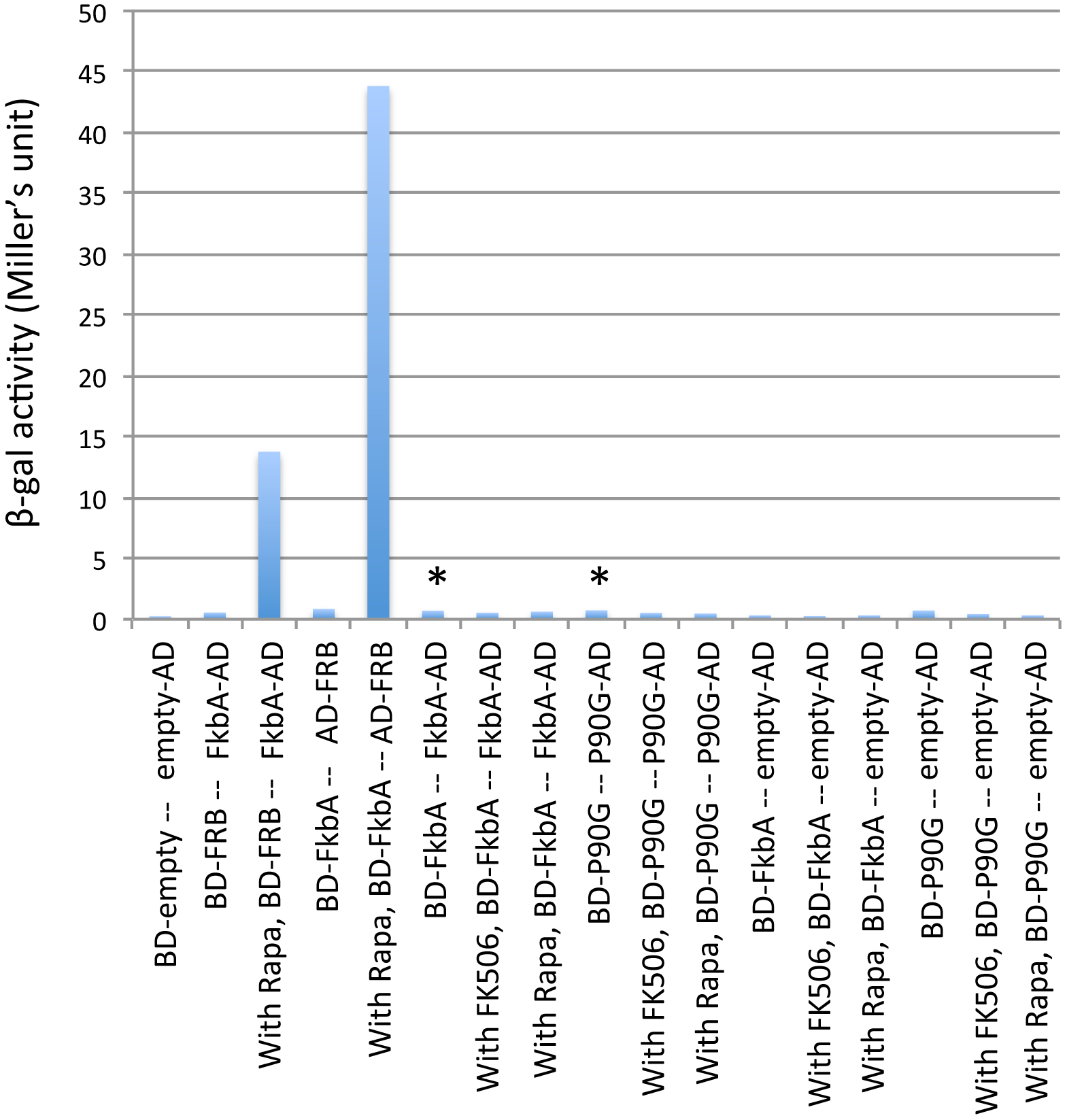

Supplement: Figure S3 — Y2H analysis for interactions between A. fumigatus FKBP12 and its variants and the FRB domain. A. fumigatus FKBP12 and its variants did not exhibit interactions based on β-galactosidase activity in the presence or absence of FK506 (1 µg/ml). The asterisks indicate that dimeric interactions were observed in vitro. In the presence of rapamycin (rapa, 1 µg/ml), FkbA-AD and BD-FkbA interact with BD-FRB and AD-FRB, respectively. Download [file mbo002162791sf3.tif]

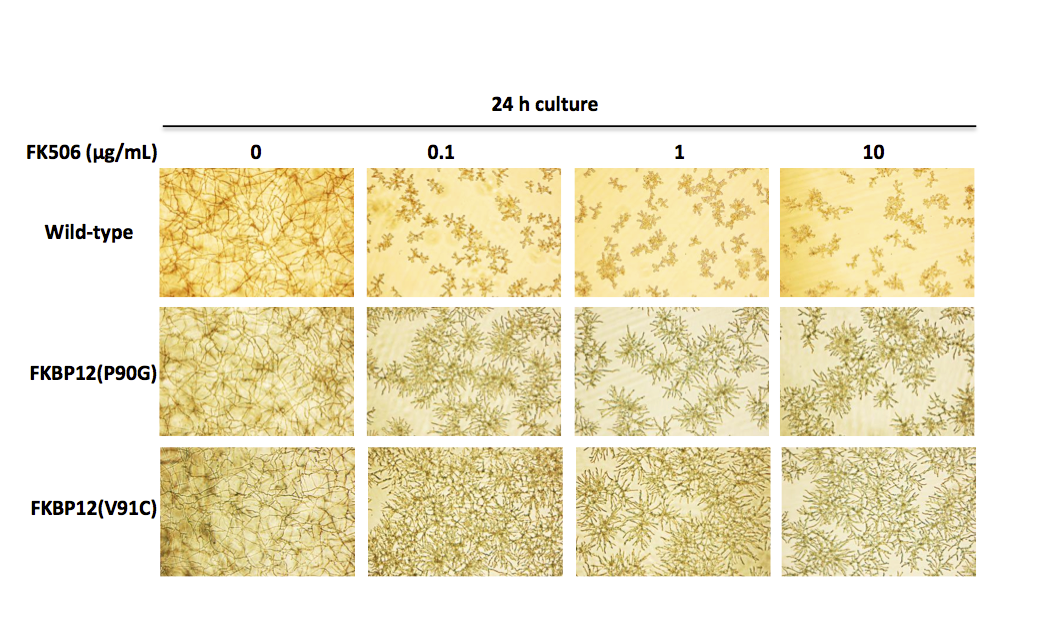

Supplement: Figure S4 — Mutations conferring FK506 resistance. The WT strain (akuBKU80) and the FKBP12(P90G)- and FKBP12(V91C)-producing strains cultured in the absence (left panel) or presence of various concentrations (0.1 to 10 µg/ml) of FK506 for 24 h were visualized for growth. A total of 1 × 10^4^ conidia of each strain were inoculated into 200 µl of RPMI liquid medium in the absence or presence of FK506, and photographs (×10 magnification) were taken after 24 h of growth. Note the slightly greater resistance of the FKBP12(V91C)-producing strain than the FKBP12(P90G)-producing strain to FK506. Experiments were repeated three times, each in triplicate. Download [file mbo002162791sf4.tif]

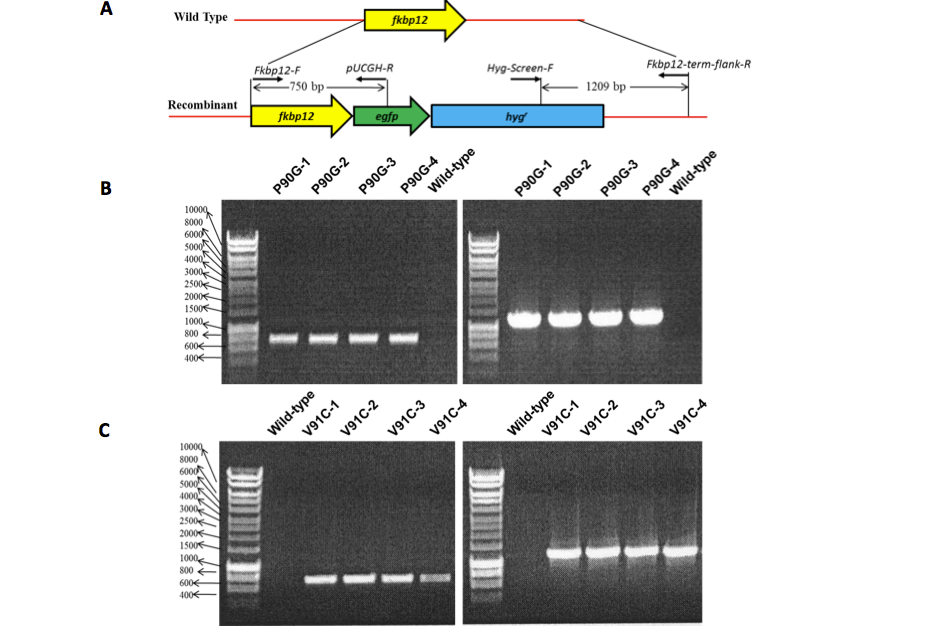

Supplement: Figure S5 — (A) Schematic representation of the genomic locus of the WT and recombinant fkbp12 mutated strains. The partial coding sequence of the A. fumigatus fkbp12 gene was replaced with the mutated version (P90G or V91C) of fkbp12 DNA fused to the gfp sequence at its C terminus with the hygromycin B resistance marker gene by homologous recombination. (B, C) PCR analysis for verification of the proper integration of the fkbp12-gfp construct at its native locus. Primers Fkbp12-F and pUCGH-R (indicated by arrows) were used to amplify the 750-bp PCR fragments from the recombinant strains (P90G and V91C transformants). Primers Hyg-Screen-F and Fkbp12-term-flank-R pUCGH-R (indicated by arrows) were used to amplify the 1,209-bp PCR fragments from the recombinant strains (P90G and V91C transformants). Download [file mbo002162791sf5.tif]
