# Supplementary material for: Structures of Pathogenic Fungal FKBP12s Reveal Possible Self-Catalysis Function
Source: mBio. 2016 Apr 26;7(2):e00492-16. doi: 10.1128/mBio.00492-16 (PMC4850266; doi:10.1128/mBio.00492-16)
Supplement: Table S1 — Plasmids and strains used for Y2H analysis. [file mbo002162791st1.doc]

Table S1. Plasmids and strains used for yeast two hybrid analysis.

|  | Genetypes or characteristics | References |
| --- | --- | --- |
| Plasmids |  |  |
| pGBKT7 | BD fusion vector, kanamycin resistance | Clontech |
| pGADT7 | AD fusion vector, ampicillin resistance | Clontech |
| pAFA1 | codon optimized *A. fumigatus* *fkbA* in pUC57 | This study |
| pAFA2 | codon optimized *A. fumigatus* *fkbA*(P90G) in pUC57 | This study |
| pAFA3 | codon optimized *A. fumigatus* *fkbA*(V91C) in pUC57 | This study |
| pAFA4 | codon optimized *A. fumigatus* FRB domain gene in pUC57 | This study |
| pAFA5 | codon optimized *A. fumigatus* *fkbA* in pGADT7, AD-FkbA fusion | This study |
| pAFA6 | codon optimized *A. fumigatus* *fkbA*(P90G) in pGAD7, AD-FkbA(P90G) fusion | This study |
| pAFA7 | codon optimized *A. fumigatus* *fkbA*(V91C) in pGADT7, AD-FkbA(V91C) fusion | This study |
| pAFA8 | codon optimized *A. fumigatus* *fkbA* in pGBKT7, BD-FkbA fusion | This study |
| pAFA9 | codon optimized *A. fumigatus* *fkbA*(P90G) in pGBKT7, BD-FkbA(P90G) fusion | This study |
| pAFA10 | codon optimized *A. fumigatus* *fkbA*(V91C) in pGBKT7, BD-FkbA(V91C) fusion | This study |
| pAFA11 | codon optimized *A. fumigatus fkbA* in pGADT7, FkbA-AD fusion | This study |
| pAFA12 | codon optimized *A. fumigatus fkbA*(P90G) in pGADT7, FkbA(P90G)-AD fusion | This study |
| pAFA13 | codon optimized *A. fumigatus fkbA*(V91C) in pGADT7, FkbA(V91C)-AD fusion | This study |
| pAFA14 | codon optimized A. fumigatus FRB domain gene in pGADT7, AD-FRB fusion | This study |
| pAFA15 | codon optimized A. fumigatus FRB domain gene in pGBKT7, BD-FRB fusion | This study |
| *S. cerevisiae* strains |  |  |
| SMY4-1 | *MATa trp1-901 his3 leu2-3, 112 ura3-52 ade2 gal4 gal80 URA3::GAL-lacZ LYS2::GAL-HIS3 TOR1-3 fpr1::ADE2* | 38 |
| AYH1 | for AD-FkbA and BD-FkbA, SMY4-1 harboring pAFA1 and pAFA4 | This study |
| AYH2 | for AD-FkbA(P90G) and BD-FkbA(P90G), SMY4-1 harboring pAFA2 and pAFA5 | This study |
| AYH3 | for AD-FkbA(V91C) and BD-FkbA(V91C), SMY4-1 harboring pAFA3 and pAFA6 | This study |
| AYH4 | for FkbA-AD and BD-FkbA, SMY4-1 harboring pAFA7 and pAFA4 | This study |
| AYH5 | for FkbA(P90G)-AD and BD-FkbA(P90G), SMY4-1 harboring pAFA8 and pAFA5 | This study |
| AYH6 | for FkbA(V91C)-AD and BD-FkbA(V91C), SMY4-1 harboring pAFA9 and pAFA6 | This study |
| AYH7 | for AD-FkbA and BD-FRB, SMY4-1 harboring pAFA1 and pAFA11 | This study |
| AYH8 | for AD-FkbA(P90G) and BD-FRB, SMY4-1 harboring pAFA2 and pAFA11 | This study |
| AYH9 | for AD-FkbA(V91C) and BD-FRB, SMY4-1 harboring pAFA3 and pAFA11 | This study |
| AYH10 | for AD-FRB and BD-FkbA, SMY4-1 harboring pAFA10 and pAFA4 | This study |
| AYH11 | for AD-FRB and BD-FkbA(P90G), SMY4-1 harboring pAFA10 and pAFA5 | This study |
| AYH12 | for AD-FRB and BD-FkbA(V91C), SMY4-1 harboring pAFA10 and pAFA6 | This study |
| AYH13 | for FkbA-AD and BD-FRBSMY4-1 harboring pAFA7 and pAFA11 | This study |
| AYH14 | for FkbA(P90G)-AD and BD-FRB, SMY4-1 harboring pAFA8 and pAFA11 | This study |
| AYH15 | for FkbA(V91C)-AD and BD-FRB, SMY4-1 harboring pAFA9 and pAFA11 | This study |
| AYH16 | for AD-FkbA and BD, SMY4-1 harboring pAFA1 and pGBKT7 | This study |
| AYH17 | for AD-FkbA(P90G) and BD, SMY4-1 harboring pAFA2 and pGBKT7 | This study |
| AYH18 | for AD-FkbA(V91C) and BD, SMY4-1 harboring pAFA3 and pGBKT7 | This study |
| AYH19 | for AD and BD-FkbA, SMY4-1 harboring pGADT7 and pAFA4 | This study |
| AYH20 | for AD and BD-FkbA(P90G), SMY4-1 harboring pGADT7 and pAFA5 | This study |
| AYH21 | for AD and BD-FkbA(V91C), SMY4-1 harboring pGADT7 and pAFA6 | This study |
| AYH22 | for FkbA-AD and BD, SMY4-1 harboring pAFA7 and pGBKT7 | This study |
| AYH23 | for FkbA(P90G)-AD and BD, SMY4-1 harboring pAFA8 and pGBKT7 | This study |
| AYH24 | for FkbA(V91C)-AD and BD, SMY4-1 harboring pAFA9 and pGBKT7 | This study |
| AYH25 | for AD and BD-FRB, SMY4-1 harboring pGADT7 and pAFA11 | This study |
| AYH26 | for AD-FRB and BD, SMY4-1 harboring pAFA10 and pGBKT7 | This study |
| AYH27 | for AD and BD, SMY4-1 harboring pGADT7 and pGBKT7 | This study |
