# Supplementary material for: Structures of Pathogenic Fungal FKBP12s Reveal Possible Self-Catalysis Function
Source: mBio. 2016 Apr 26;7(2):e00492-16. doi: 10.1128/mBio.00492-16 (PMC4850266; doi:10.1128/mBio.00492-16)
Supplement: Table S2 — Primers used to generate mutated FKBP12-GFP-producing strains. [file mbo002162791st2.doc]

**Table S2: Primers Used to Generate Mutated FKBP12-GFP Strains**

| **Name** | **Sequence (5’-3’)** | **Direction** |
| --- | --- | --- |
| ***FKBP12-P90G-GFP*** | CAAGGGTACCTTATCGTGATCC  TGGGTTCCCTGGCGTCATCCC  GGGATGACGCCAGGGAACCCA  ATGCGGATCCAGCCCTCTTGTTGTTGATG | Forward  Forward  Reverse  Reverse |
| Fkbp12-1-gene-F-KpnI-gfp Fkbp12-P90G-F  Fkbp12-P90G-R  Fkbp12-1-gene-R-Bam-H1-gfp |
| ***FKBP12-V91C-GFP*** | CGACGGATCCATGGATCAAGCACTGGCG TGGGTTCCCTCCCTGCATCCCTG  CAGGGATGCAGGGAGGGAACCCA  CGACGGATCCGGCTTCCCTAGTCTC | Forward  Forward  Reverse  Reverse |
| Fkbp12-1-gene-F-KpnI-gfp Fkbp12-V91C-F  Fkbp12-V91C-R  Fkbp12-1-gene-R-Bam-H1-gfp |
| ***For PCR verification*** | ATGGGTGTCACCAAGGAACT  aagtcctcgtgtactgtgtaagcg  acacgctgaacttgtg  GATTGTTGGATGCTGAAGATGG | Forward  Forward  Reverse  Reverse |
| Fkbp12-F  Hyg-Screen-F-New  pUCGH-R  FKBP12-1-term-flank-R |
| ***For Sequencing*** | Gcgttggccgattcatta  acacgctgaacttgtg | Forward  Reverse |
| pUCGH-2033-F  pUCGH-R |
